# Serum Magnesium and Cognitive Function Among Qatari Adults

**DOI:** 10.3389/fnagi.2020.00101

**Published:** 2020-04-15

**Authors:** Kateba Al-Ghazali, Sana Eltayeb, Ayesha Musleh, Tamara Al-Abdi, Vijay Ganji, Zumin Shi

**Affiliations:** Human Nutrition Department, College of Health Sciences, QU Health, Qatar University, Doha, Qatar

**Keywords:** serum magnesium, mean reaction time, cognition, qatar biobank, adults, cross-sectional study

## Abstract

**Background**: Previous studies found that low blood magnesium increases the risk of several diseases such as cardiovascular diseases (CVD), diabetes, and hypertension. These ailments are associated with declined cognitive function.

**Objective**: We aimed to examine the association between serum magnesium and cognitive function among Qatari adults. In addition, we assessed the interaction relation between low serum magnesium, hypertension, and diabetes in relation to cognitive function.

**Method**: Data from 1,000 Qatari participants aged ≥20 years old who participated in the Qatar Biobank (QBB) Study were analyzed. Serum magnesium was measured by an automated calorimetric method and suboptimal magnesium was characterized by <0.85 mmol/l. Cognitive function was determined by measuring the mean reaction time (MRT) based on a computer-based, self-administered test. Multivariable linear regression was used to examine the relation between serum magnesium concentrations and cognitive function.

**Results**: The prevalence of suboptimal magnesium was 57.1%. Across the four quartiles of serum magnesium from the lowest to the highest, the regression coefficients (95% CI) for MRT were 0 (reference), −17.8, −18.3, and −31.9 (95% CI 2.4–3.1; *p* for trend 0.05). The presence of hypertension and diabetes significantly increased the MRT. Women with suboptimal magnesium and hypertension had the highest MRT.

**Conclusion**: The prevalence of suboptimal magnesium is high in Qatar. There was a direct association between serum magnesium and cognitive function. Low magnesium concentrations were associated with a longer MRT.

## Introduction

Dementia is a major neurocognitive disorder characterized by a decline in memory, problem-solving, language, and other cognitive skills (Hugo and Ganguli, 2014). According to the World Alzheimer Report 2018, 50 million people are living with dementia and it will triple to 152 million by 2050 (Alzheimer’s Disase International, 2018). Nutrition is an important modifiable factor that affects cognitive function (Scarmeas et al., 2018). Most of the existing studies found a beneficial effect of fish, nuts, and fresh vegetable intake on cognition (Scarmeas et al., 2018). However, the association between individual nutrients intake and cognition is inconclusive (Scarmeas et al., 2018).

Magnesium is one of the most abundant cations in the human body (Jahnen-Dechent and Ketteler, 2012). It is a cofactor for more than 300 enzymatic reactions. These enzymes function in regulating blood pressure, controlling blood glucose, and synthesis of several biomolecules such as proteins, DNA, and RNA (Gröber et al., 2015). Moreover, magnesium plays a role in neuromuscular conduction and nerve-impulse transmission. It also has a protective role against neuronal cell death (Kirkland et al., 2018). Therefore, magnesium may have a potential role in neurological disorders (Kirkland et al., 2018).

Magnesium can be found in various food sources such as nuts, legumes, dairy products, and green leafy vegetables. In modern society with a high consumption of refined grains and low intake of vegetables, the intake of magnesium is low (Costello R. et al., 2016). Serum concentrations of magnesium is used as a marker of magnesium nutritional status. Studies have shown that low magnesium can increase the risk of several diseases such as cardiovascular diseases (CVD; Fang et al., 2016; Azab et al., 2019), diabetes mellitus, and hypertension (Costello R. et al., 2016). These diseases are associated with declined cognitive function (Biessels and Despa, 2018).

Evidence from animal studies supports the role of magnesium in cognitive function. For example, a study conducted by Xu et al. (2014) found that magnesium protects cognitive function and synaptic plasticity by inhibiting GSK-3β in a rat model with Alzheimer’s disease. However, human studies on magnesium and cognition are scarce across the world. Higher dietary magnesium intake was associated with a reduced risk of developing cognitive impairment in an 8-year follow-up study in Australia (Cherbuin et al., 2014). The findings are supported by the fact that Alzheimer’s disease patients have lower plasma magnesium concentrations (Barbagallo et al., 2011). In stroke patients, low serum magnesium concentrations were associated with post-stroke cognitive impairment (Tu et al., 2018). However, the relation between magnesium and cognitive function in the general population is not clear.

A recent study on 9,693 Qatari adults found that subclinical magnesium deficiency (serum magnesium <0.85 mmol/l) is common. Low circulating magnesium is positively associated with prediabetes, diabetes, and hypertension in Qatari adults (Shi and Abou-Samra, 2019). However, no studies have assessed the relationship between serum magnesium and cognition in Qatar, where the prevalence of diabetes is about ~19% (Al-Thani et al., 2017a). Therefore the objectives of this study were to determine the association between serum magnesium and cognitive function and to assess the interaction relation between low serum magnesium, hypertension, and diabetes in relation to cognitive function in Qatari adults.

## Materials and Methods

### Study Population

Adults aged 20 years and above were recruited by Qatar Biobank (QBB). They were Qatari nationals or were living in the country for more than 15 years. These subjects were followed up for every 5 years. The details of the study design were published elsewhere (Al Kuwari et al., 2015). Sociodemographic information, lifestyle factors, and dietary habits were collected using a self-administered questionnaire. Whereas a nurse interview was used to collect information on health conditions, family history of the disease, and medication use. Each participant underwent a health examination at Hamad Medical Center, Doha.

Body height and weight were measured using a Seca stadiometer. Blood samples (60 ml) were taken and measured for a total of 66 biomarkers at the QBB facility. A random sample of 1,000 Qatari participants aged 20 years and older were included in the current analysis. All the participants had measured serum magnesium concentration and underwent a cognitive function test.

### Outcome Variable: Cognitive Function (Mean Reaction Time)

Mean reaction time (MRT) was used as an indicator of cognitive function. It was measured by a computer-based, self-administered touch screen test comprising 60 tasks (Al Kuwari et al., 2015; Lyall et al., 2016). The stimulus used for the assessment of MRT was visual. In addition to MRT, a paired episodic memory test was conducted. However, due to the limited variation of the test results, the memory test was not included in the current study.

### Exposure Variable: Serum Magnesium

Serum Magnesium was assessed by an automated colorimetric method (Magnesium Gen.2 from Roche Diagnostics, Indianapolis, IN, USA) in the central lab of QBB (Costello R. B. et al., 2016). The coefficients of variations are 0.3–0.8%. Subclinical magnesium deficiency was characterized as serum magnesium <0.85 mmol/l.

### Covariates

Gender, age, education (low: without university degree; high: with a university degree), smoking (non-smokers, ex-smokers, and current smokers), leisure-time physical activity level [the metabolic equivalent of task (MET), recoded as tertiles], and BMI (overweight was defined as a BMI of 25.0–29.9 kg/m^2^ and obesity was defined as a BMI of ≥30 kg/m^2^; Al-Thani et al., 2019) were used as covariates. The diagnostic criteria for assessing diabetes were: HbA1c ≥6.5%, random blood glucose ≥11.1 mmol/l, fasting blood glucose ≥7 mmol/l, or self-reported diabetes (American Diabetes Association, 2014). Hypertension was diagnosed as systolic blood pressure >140 mmHg and/or diastolic blood pressure >90 mmHg or previous doctor diagnosis.

### Data Analysis

The serum magnesium concentrations were stratified into gender-specific quartiles. Mean (SD) or percentage were used to present the sample characteristics. To compare the differences between quartile sample characteristics, a Chi-squared test was used for categorical variables and ANOVA was used for continuous variables. To assess the association between serum magnesium and MRT, three multivariable linear regression models were used. Model 1 was adjusted for gender and age (continuous), Model 2 was further adjusted for smoking, education, BMI, and physical activity. Model 3 was further adjusted hypertension, diabetes and the for use of medication for hypertension and diabetes. The interaction between serum magnesium, cognitive function, and chronic diseases (diabetes and hypertension) was tested by adding the product terms of the variables in the linear regression model. To visualize the interaction, a command *(marginsplot)* in Stata was used. All the analyses were conducted using STATA (Version 16, Stata Corporation, College Station, TX, USA). We considered *p*-values < 0.05 (2-tailed) as statistically significant.

## Results

### Sample Description

The mean age of the sample was 35.8 (SD 10.3) years. The mean serum magnesium concentration was 0.84 (SD 0.08) mmol/l. The mean MRT was 715.3 (SD 204.1) milliseconds. Overall, 57.1% of participants had a subclinical magnesium deficiency. More than half of the participants were highly educated and 33.9% have low education levels. 67.3% of subjects were non-smokers, 18.7% were smokers and 14% were ex-smokers. Overall, leisure-time physical activity was low. The majority of the sample was overweight (38.2%) or obese (32.5%). A total of 61.4% of the sample reported using supplements. 38.3% were using vitamin D and calcium supplements. Supplementary Figure S1 shows the distribution of serum magnesium concentrations among the study sample. Most of the participants had concentrations between 0.75–0.9 mmol/l.

Across the quartiles of serum magnesium from the lowest to the highest, there was no difference in age, gender, education, and smoking (Table 1). However, insulin use, diabetes oral medication, and hypertension medication were higher among people who have low serum magnesium. Age and female gender were associated with a higher MRT while education was associated with a lower MRT (Supplementary Figure S2). No association was observed between MRT and smoking and physical activity.

**Table 1:** Sample characteristics by quartiles of serum magnesium concentrations^1^.

	Q1	Q2	Q3	Q4	*p*-value
	*N* = 294	*N* = 242	*N* = 237	*N* = 227	
Magnesium (mmol/l)	0.77 (0.03)	0.82 (0.01)	0.86 (0.01)	0.91 (0.03)	<0.001
Age (years)	36.1 (10.3)	35.3 (10.3)	35.9 (10.7)	36.1 (10.0)	0.80
Gender					0.24
Male	152 (51.7%)	113 (46.7%)	129 (54.4%)	106 (46.7%)
Female	142 (48.3%)	129 (53.3%)	108 (45.6%)	121 (53.3%)	
Education					0.32
Low	105 (35.7%)	73 (30.3%)	88 (37.3%)	72 (31.7%)	
High	189 (64.3%)	168 (69.7%)	148 (62.7%)	155 (68.3%)
Smoking status					0.56
Non-smoker	192 (65.3%)	161 (66.5%)	158 (66.7%)	162 (71.4%)	
Current Smoker	53 (18.0%)	45 (18.6%)	48 (20.3%)	41 (18.1%)	
Ex-smoker	49 (16.7%)	36 (14.9%)	31 (13.1%)	24 (10.6%)	
Leisure time physical activity (MET h/week)	7.9 (32.3)	4.5 (12.8)	7.0 (20.6)	5.2 (16.4)	0.30
BMI (kg/m^2^)	28.8 (5.5)	28.3 (5.7)	27.6 (5.9)	28.0 (5.8)	0.11
BMI categories					0.084
Normal	69 (23.5%)	71 (29.3%)	85 (35.9%)	68 (30.0%)	
Overweight	120 (40.8%)	87 (36.0%)	86 (36.3%)	89 (39.2%)	
Obese	105 (35.7%)	84 (34.7%)	66 (27.8%)	70 (30.8%)
Supplement use	188 (63.9%)	143 (59.1%)	145 (61.2%)	138 (60.8%)	0.71
Vitamin D and Calcium supplements use	126 (42.9%)	93 (38.4%)	84 (35.4%)	80 (35.2%)	0.23
HbA1C (%)	5.7 (1.3)	5.5 (0.8)	5.5 (0.7)	5.5 (0.6)	0.002
Hypertension	33 (11.2%)	18 (7.4%)	24 (10.1%)	21 (9.3%)	0.51
Diabetes	46 (16.2%)	27 (11.6%)	21 (9.2%)	22 (10.2%)	0.069
Insulin use	13 (4.4%)	3 (1.2%)	3 (1.3%)	0 (0.0%)	0.001
Diabetes medication use other than insulin	29 (9.9%)	10 (4.1%)	8 (3.4%)	8 (3.5%)	0.002
Hypertension medication use	26 (8.8%)	10 (4.1%)	10 (4.2%)	9 (4.0%)	0.030
Mean reaction time (millisecond)	732 (218)	710 (198)	711 (215)	703 (178)	0.38

*^1^Values are mean (SD) or n (%)*.

### Association Between Serum Magnesium With MRT

Serum magnesium concentration was inversely associated with MRT. After adjusting for sociodemographic characteristics, lifestyle factors, across the quartiles of serum magnesium, the regression coefficients (95% CI) for MRT were 0 (reference), −17.8 (2nd quartile), −18.3 (3rd quartile), and −31.9 (4th quartile; *p* for trend 0.05; Table 2). Further adjusting for hypertension, diabetes, and medication use, the above association was attenuated and became statistically non-significant. Similarly, using serum magnesium as a continuous variable, there was an inverse relationship between magnesium and MRT before the adjustment of medication use.

**Table 2:** Association between serum magnesium concentrations and cognitive function^1^.

	Quartiles of serum magnesium						
	Q1	Q2	Q3	Q4	*p* for trend	Magnesium (continuous)	*p*
Model 1^2^	0 (reference)	−19.9 (−50.9 to 11.1)	−16.4 (−47.5 to 14.8)	**−34.1 (−65.6 to −2.54)**	0.049	**−198.9 (−391.4 to −6.3)**	0.043
Model 2^3^	0 (reference)	−17.8 (−48.4 to 12.8)	−18.3 (−49.0 to 12.5)	**−31.9 (−63.0 to −0.82)**	0.05	**−206.3 (−396.1 to −16.4)**	0.033
Model 3^4^	0 (reference)	−9.3 (−40.4 to 21.8)	−10.7 (−42.2 to 20.8)	−24.7 (−56.5 to 7.2)	0.14	−140.1 (−336.2 to 56.1)	0.16

*^1^Values are regression coefficients (95% CI) from multivariable linear regression. ^2^Adjusted for age and gender. ^3^Further adjusted for education, smoking, and physical activity. ^4^Further adjusted for BMI, diabetes, hypertension, and medication for diabetes and hypertension. *p values* < 0.05*.

**Figure 1:**
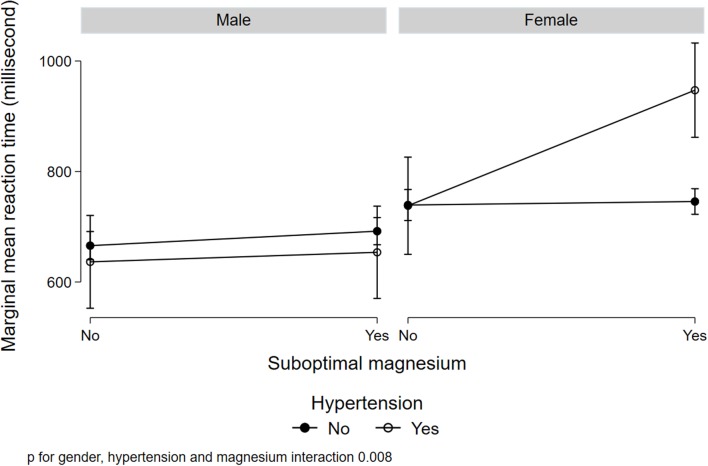
Interaction between serum magnesium concentrations, hypertension, and gender with cognition. Values represent the adjusted mean (SD) of mean reaction time (MRT) derived from multivariable linear regression. Model adjusted for gender, age, education, BMI, smoking status, leisure-time physical activity, diabetes, hypertension, and current medication use for hypertension and diabetes.

**Figure 2:**
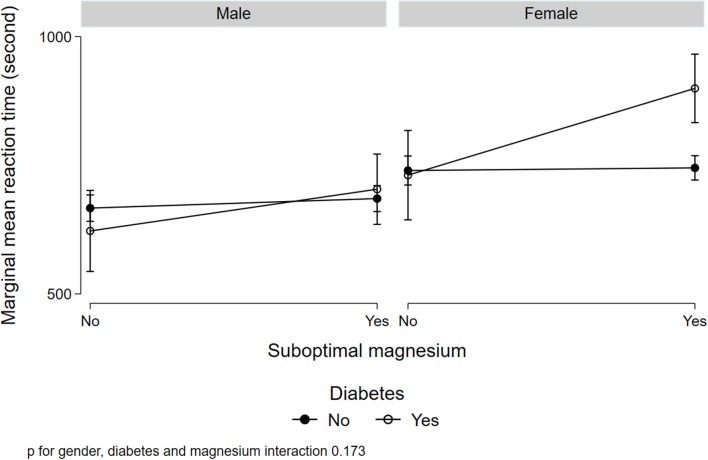
Interaction between serum magnesium concentrations, diabetes, and gender with cognition. Values represent the adjusted mean (SD) of MRT derived from multivariable linear regression. Model adjusted for gender, age, education, BMI, smoking status, leisure-time physical activity, diabetes, hypertension, and current medication use for hypertension and diabetes.

**Figure 3:**
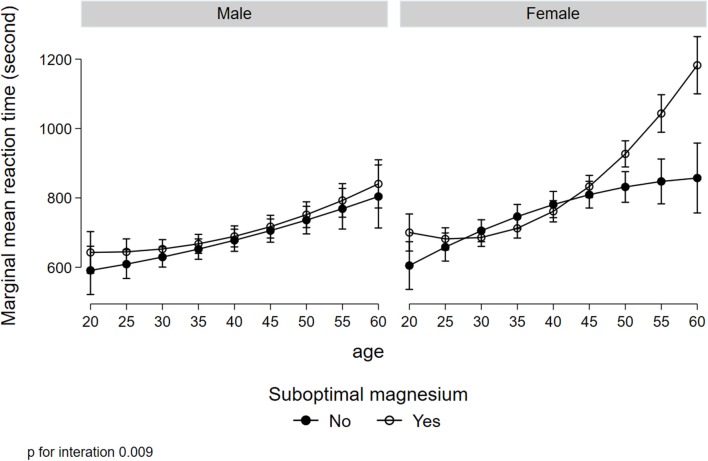
Interaction between gender, suboptimal serum magnesium, and age in relation to MRT. Values represent the adjusted mean (SD) of MRT derived from multivariate linear regression. Model adjusted for gender, age, education, BMI, smoking status, leisure-time physical activity, diabetes, hypertension, and current medication use for hypertension and diabetes.

Figure 1 illustrated a significant interaction (*p* = 0.008) between gender, low serum magnesium, and hypertension in relation to MRT. Women with suboptimal magnesium and hypertension had the highest MRT. Although a three-way interaction between gender, suboptimal magnesium, and diabetes was not statistically significant (*p* = 0.173), women with suboptimal magnesium and diabetes had the highest MRT (Figure 2). There was a significant interaction between suboptimal magnesium, gender, and age in relation to MRT (Figure 3). Among women with suboptimal magnesium, there was an abrupt increase in the MRT when the age was above 40 years old. No such association was observed in men.

## Discussion

In this cross-sectional study, serum magnesium concentration was inversely associated with MRT. Low serum magnesium was prevalent in Qatari adults and was associated with longer MRT. There was a significant interaction between low serum magnesium, hypertension, and gender in relation to MRT. There was also an interaction between gender, diabetes, and low serum magnesium. Those with hypertension and low serum magnesium concentrations, the MRT was longer in women but not in men.

Our findings are consistent with previous studies on the association between serum magnesium concentrations and cognition in both animal and human studies (Allaert et al., 2016; Serefko et al., 2016; Balmus et al., 2017; Boyle et al., 2017; Iolascon et al., 2017; Kirkland et al., 2018). In a case-control study of 30 patients (15 with Alzheimer’s disease and 15 with mild cognitive impairment) and 15 control participants, lower magnesium concentrations were found among patients with Alzheimer’s disease and mild cognitive impairment (Balmus et al., 2017). Evidence from animal studies supports a link between circulating magnesium and cognition (Slutsky et al., 2010; Li et al., 2014; Huang et al., 2018; Tu et al., 2018). Low magnesium impairs cognition (Tu et al., 2018) and magnesium supplementation improves cognition in several studies (Slutsky et al., 2010; Li et al., 2014; Huang et al., 2018). For example, mice fed with a magnesium-deficient diet showed impairments in the three types of hippocampus-dependent memory including contextual, social recognition, and spatial memories (Noble et al., 2014). In contrast, rats with hepatic encephalopathy after administration of oral magnesium showed an improvement in cognitive and locomotor functions by reducing the brain manganese concentration and regulating glutamine synthetase (Han et al., 2019).

Human studies on the relationship between magnesium intake and cognition are limited (Iranpour et al., 2019). Data from the National Health and Nutrition Examination Survey (NHANES) showed that magnesium intake was directly associated with improved cognitive function markers such as Digital Symbol Substitution, Animal Fluency tests, immediate recall score, and delayed recall score (Iranpour et al., 2019). Diets rich in magnesium have shown to be positively associated with cognition (Shakersain et al., 2018). A study conducted in Sweden found that subjects who consumed a Nordic Prudent diet rich in magnesium were associated with an improved cognitive function (Shakersain et al., 2018). A prospective cohort study in New York (*n* = 2148) suggested that Alzheimer’s Disease risk could be decreased with the adherence to a dietary pattern characterized by high consumption of magnesium-rich foods such as fish, vegetables, and fruits (Gu et al., 2010). Findings from clinical trials also support the beneficial effects of magnesium on cognition (Cohen-Hagai et al., 2018). In a recent prospective, randomized, double-blinded study (*n* = 22), a positive association between serum and intracellular magnesium and cognitive performance was observed. Also, magnesium deficiency was associated with impaired cognition (Cohen-Hagai et al., 2018). In a 12-week randomized, double-blind, placebo-controlled, parallel designed trial (*n* = 44), magnesium has been shown to have the potential for treating cognitive impairment (Liu et al., 2016).

The interaction between gender, hypertension/diabetes, and low serum magnesium is intriguing. Although the mechanisms are not clear, maintaining adequate circulating magnesium seems to be especially important in women. Consistent with previous studies (Hyde, 2016), in this study, women have lower MRT compared to men. Further studies are needed to elucidate the mechanisms linking magnesium with cognition. Further clinical trials are warranted to test the efficacy of magnesium supplementation in cognition in women with diabetes and hypertension.

Several mechanisms may explain the association between magnesium and cognition. First, adequate magnesium is essential for the prevention and management of diabetes and hypertension. Diabetes and hypertension (Tadic et al., 2016; Biessels and Despa, 2018) are risk factors for cognitive impairment. In Qatar, the diabetes burden is among one of the highest in the world (Al-Thani et al., 2017a). Second, magnesium has antioxidant properties and can reduce the levels of oxidative stress and inflammation (Zheltova et al., 2016). The latter plays an important role in brain aging and cognitive function decline (Walker et al., 2019).

The causes of low serum magnesium in this study may be multifactorial. Diabetes and hypertension can be significant contributors to hypomagnesemia. In Qatar, the prevalence of diabetes was ≈19% among adults aged 18–64 years old (Al-Thani et al., 2017a). The contribution of diet on serum magnesium levels seems to be small based on a previous study in Qatar. A dietary pattern using a reduced rank regression method with serum magnesium as the response variable can only explain 0.9% of the variance of serum magnesium (Shi and Abou-Samra, 2019). In Qatar, the main source of drinking water is desalinated seawater. Magnesium concentration in desalinated water is significantly lower than in non-desalinated municipal water. For example, desalinated water contained 10.9 mg/l compared to 18 mg/l in other bottled water (Rowell et al., 2015). It is not known to what extent the consumption of desalinated seawater contributes to the low serum magnesium in Qatar.

On the other hand, based on the dietary patterns in Qatar, refined cereals/grains were the second most consumed product in Qatar. Refined grains tend to be low in magnesium. Moreover, Qataris consume more oils, fats, sugar, and sweets than non-Qataris (Al-Thani et al., 2017b). This may also contribute to lower magnesium dietary intake. Further studies are needed to identify the contributors of serum magnesium. With the population aging rapidly, in combination with the high burden of chronic diseases, intervention is needed to prevent cognitive function decline.

Our study has several strengths. First, the participants were from the general population in Qatar. Second, because of the richness of QBB data, we were able to adjust the analysis for various potential confounding variables. Because of the cross-sectional study design, the results should not be viewed in terms of cause and effect. Another limitation was that the cognitive function test included only MRT. As QBB started to conduct an MRI brain scan, it will allow us to examine the relationship between serum magnesium and brain image in the future. Furthermore, the mean age of the sample was young. It may not represent the Qatar population at-large.

## Conclusion and Future Work

There was a direct association between serum magnesium and cognitive function. Low magnesium concentrations were associated with a longer MRT. Women with diabetes or hypertension were affected the greatest by low magnesium concentrations. Therefore, they may derive benefits from the consumption of magnesium-rich foods such as nuts, fish, leafy vegetables, and legumes. Further longitudinal studies and clinical trials are needed to verify whether magnesium intake and supplementation may improve cognitive function in the population.

## Data Availability Statement

The dataset generated for this study are available on request to the Qatar Biobank Study data management team.

## Ethics Statement

The QBB study was reviewed and approved by the Institutional Review Board (IRB) from the Hamad Medical Corporation Ethics Committee. All participants gave written informed consent before participation. The current study was approved under the IRB exempted category (Ex-2019-RES-ACC-0163-0086) by the QBB.

## Author Contributions

ZS: conceptualization, formal analysis, resources, supervision, and project administration. ZS, VG, KA-G, SE, AM, and TA-A: methodology and writing—review and editing. ZS, VG, KA-G, SE, AM, and TA-A: writing—original draft preparation. All authors read and approved the final version of the manuscript.

## Conflict of Interest

The authors declare that the research was conducted in the absence of any commercial or financial relationships that could be construed as a potential conflict of interest.
