# Supplementary material for: Serum Magnesium and Cognitive Function Among Qatari Adults
Source: Front Aging Neurosci. 2020 Apr 15;12:101. doi: 10.3389/fnagi.2020.00101 (PMC7174684; doi:10.3389/fnagi.2020.00101)
Supplement: Supplementary file 1 [file Data_Sheet_1.docx]

Supplementary Material

**Supplement Figure 1** Distribution of serum magnesium levels

**Supplement Figure 2** Association between sociodemographic factors, lifestyle, serum magnesium and mean reaction time

Values were regression coefficients (95%CI) for MRT adjusting for all the variables in the figure.
